# Supplementary material for: Modelling spatiotemporal patterns of visceral leishmaniasis incidence in two endemic states in India using environment, bioclimatic and demographic data, 2013–2022
Source: PLoS Negl Trop Dis. 2024 Feb 5;18(2):e0011946. doi: 10.1371/journal.pntd.0011946 (PMC10868833; doi:10.1371/journal.pntd.0011946)

S1 Fig. Maps of covariates (averaged for the period 2013-2020) used in models. (A) Monthly mean temperature per month (BIO1, °C), (B) Isothermality (BIO3, %), (C) average precipitation per month (BIO12, mm), (D) Monthly maximum temperature (°C), (E) Monthly minimum temperature (°C), (F) Soil moisture (m^3^/m^3^), (G) Population density (per Km^2^), (H) Enhanced vegetation index (spectral index), (I) Land Surface Temperature (°C)

1.
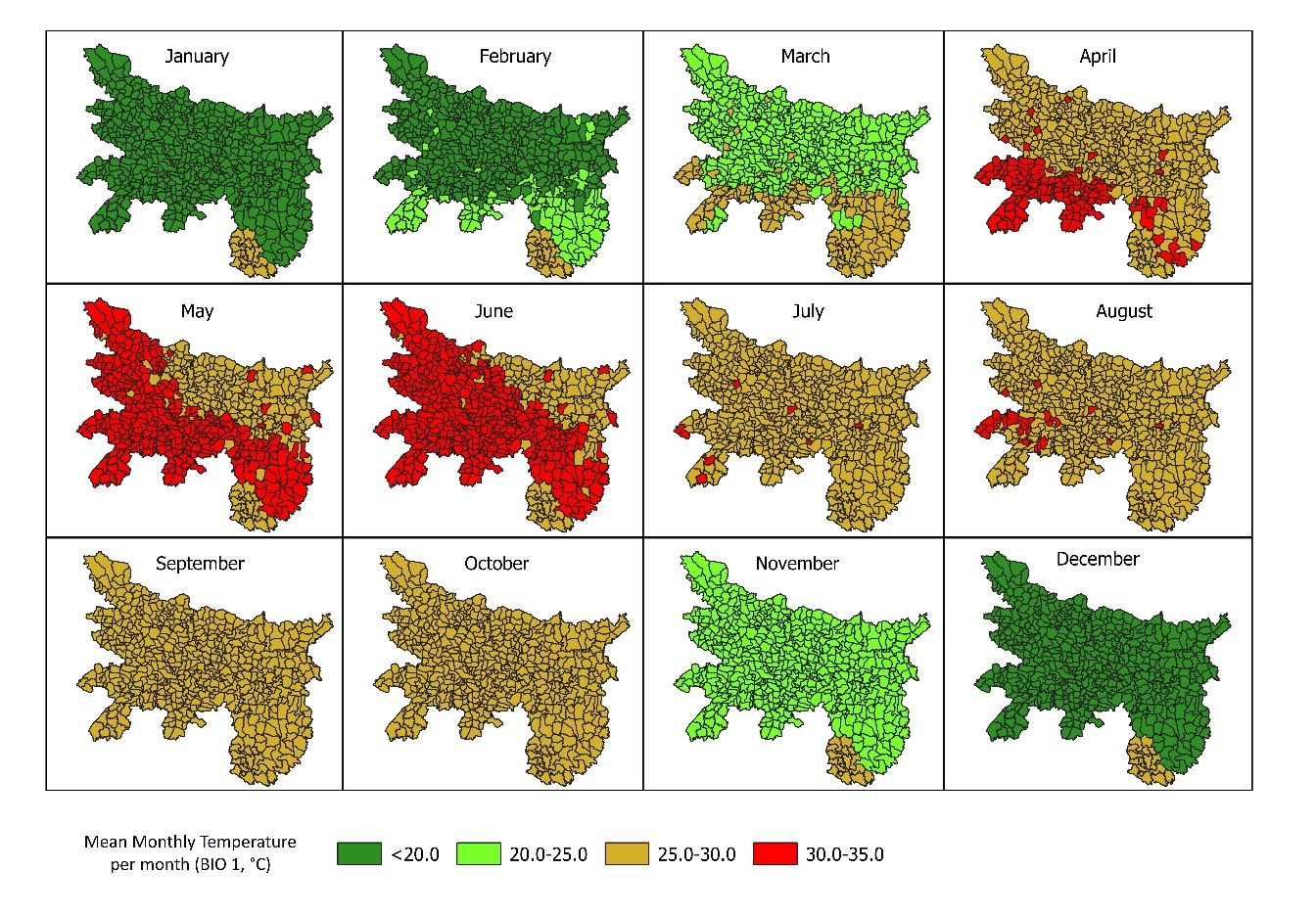
**Monthly mean temperature per month (BIO1, °C)**
2.
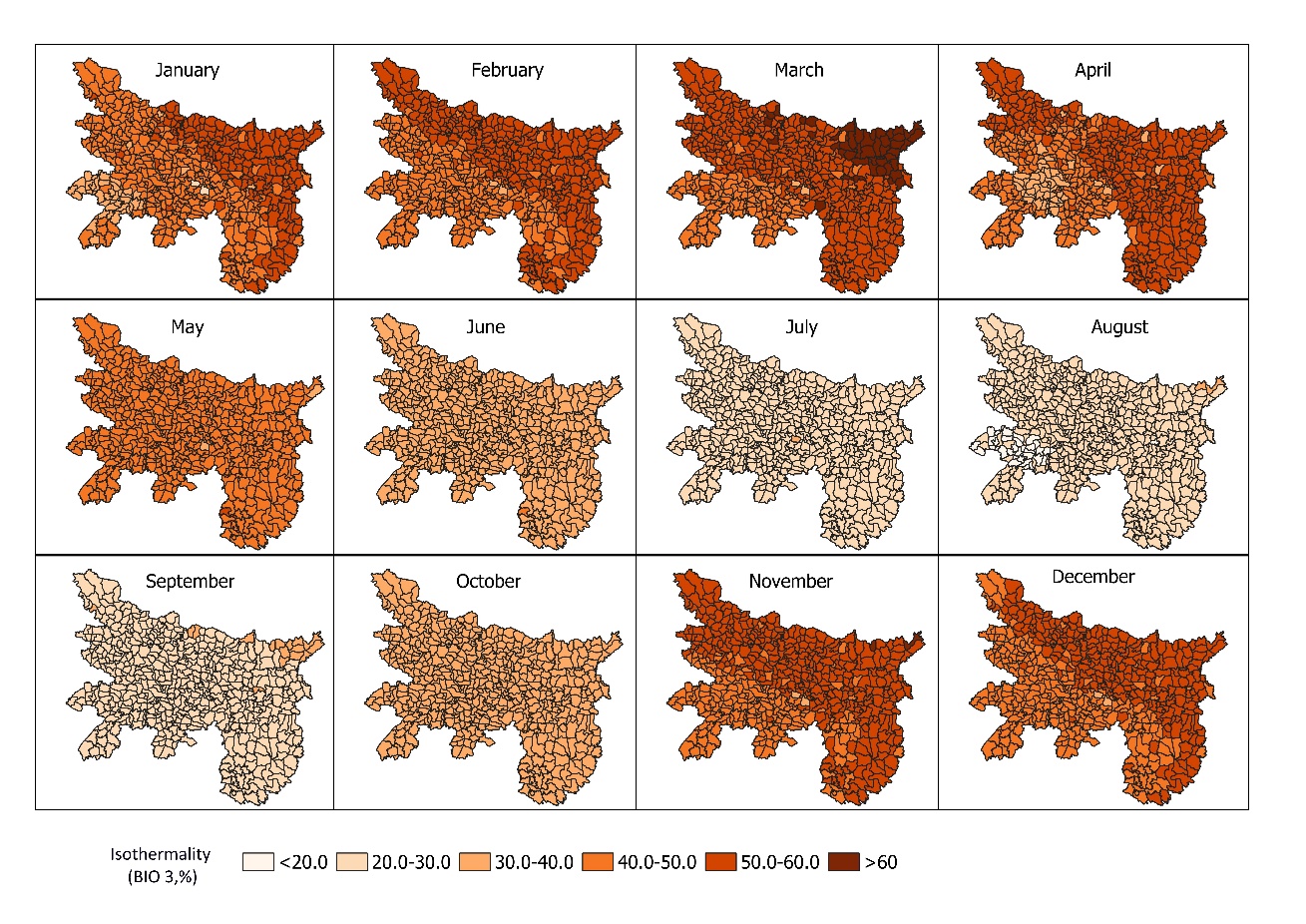
**Isothermality (BIO3, %)**
3.
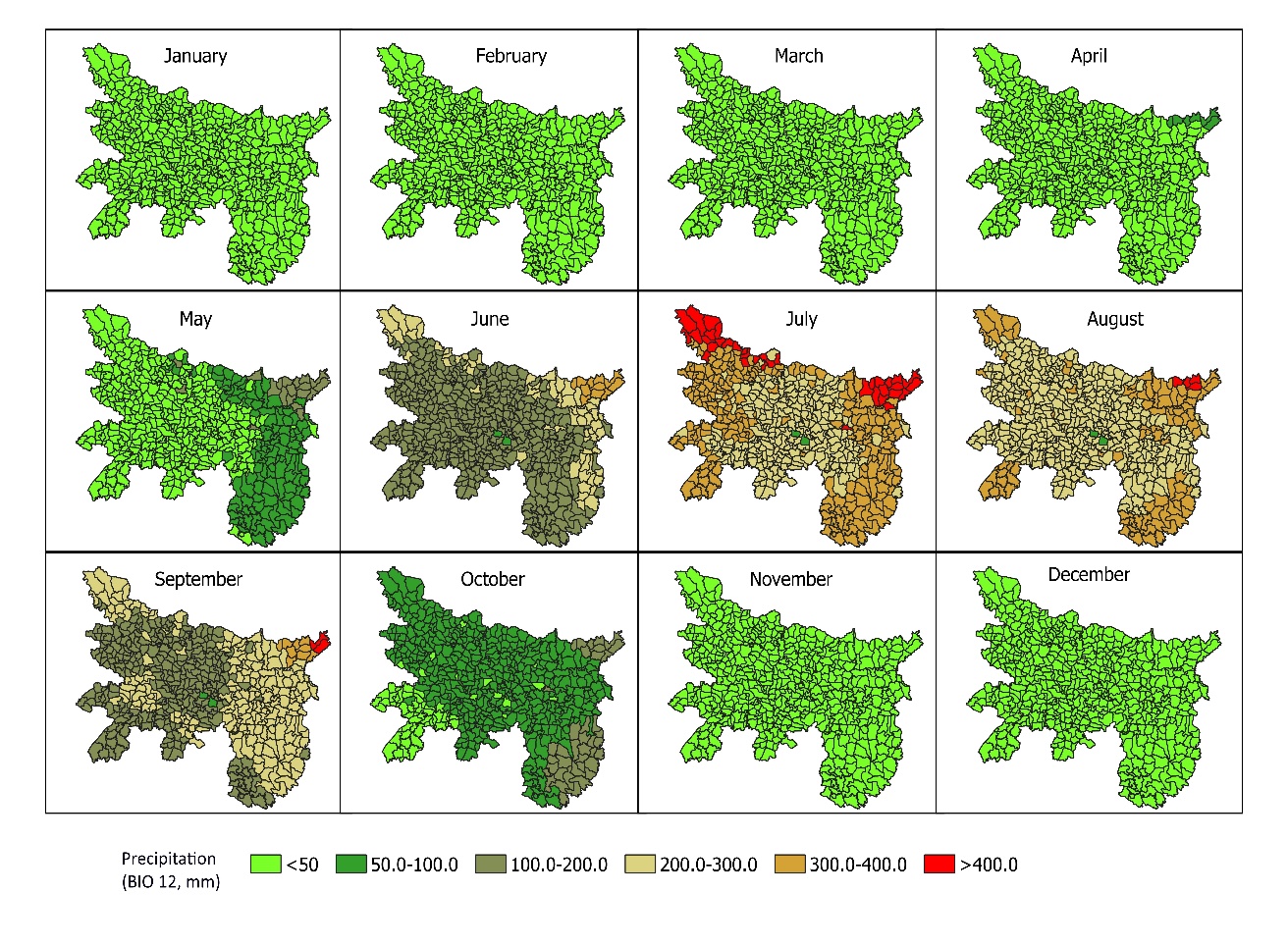
Monthly average precipitation (BIO-12, mm)
4.
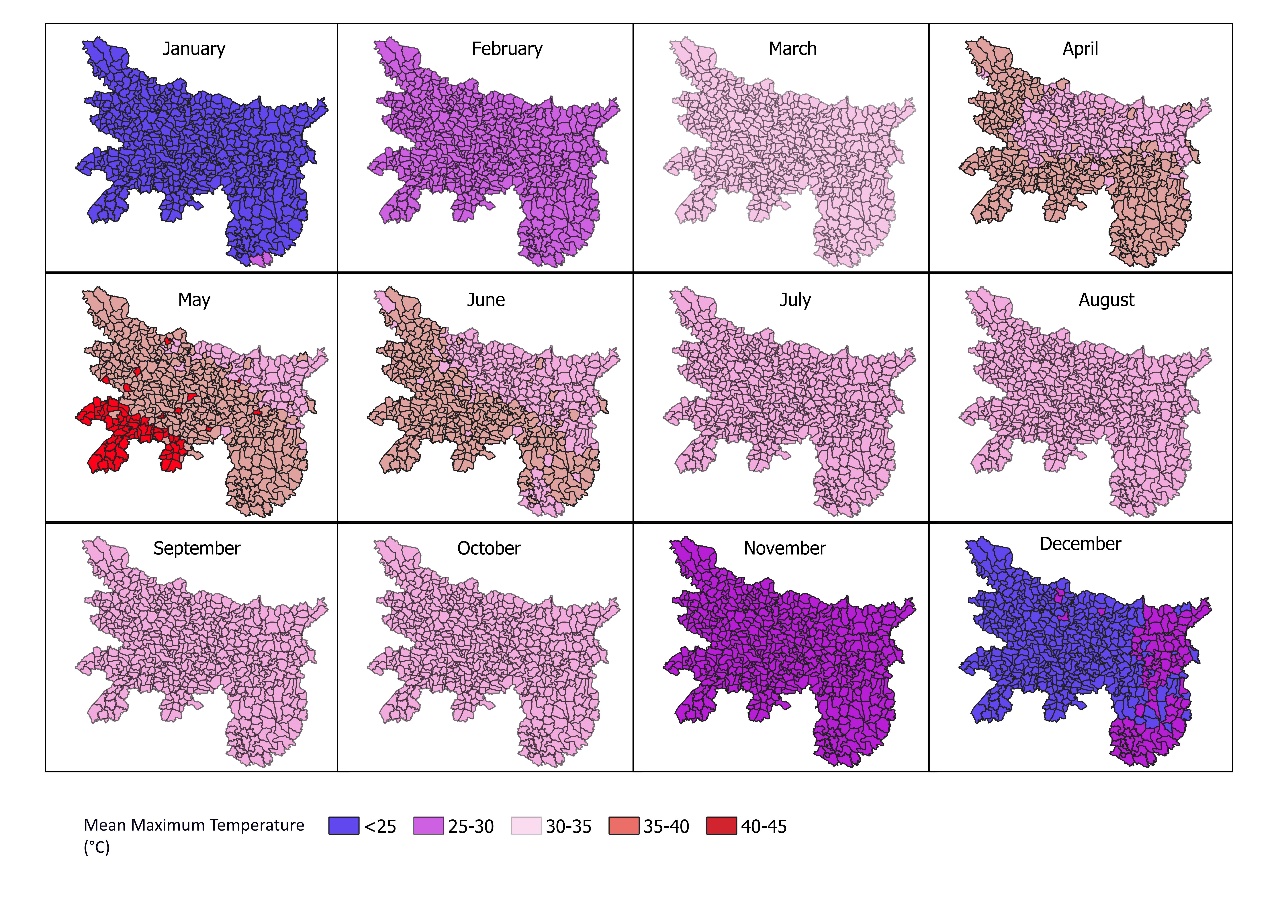
**Monthly maximum temperature (°C)**
5.
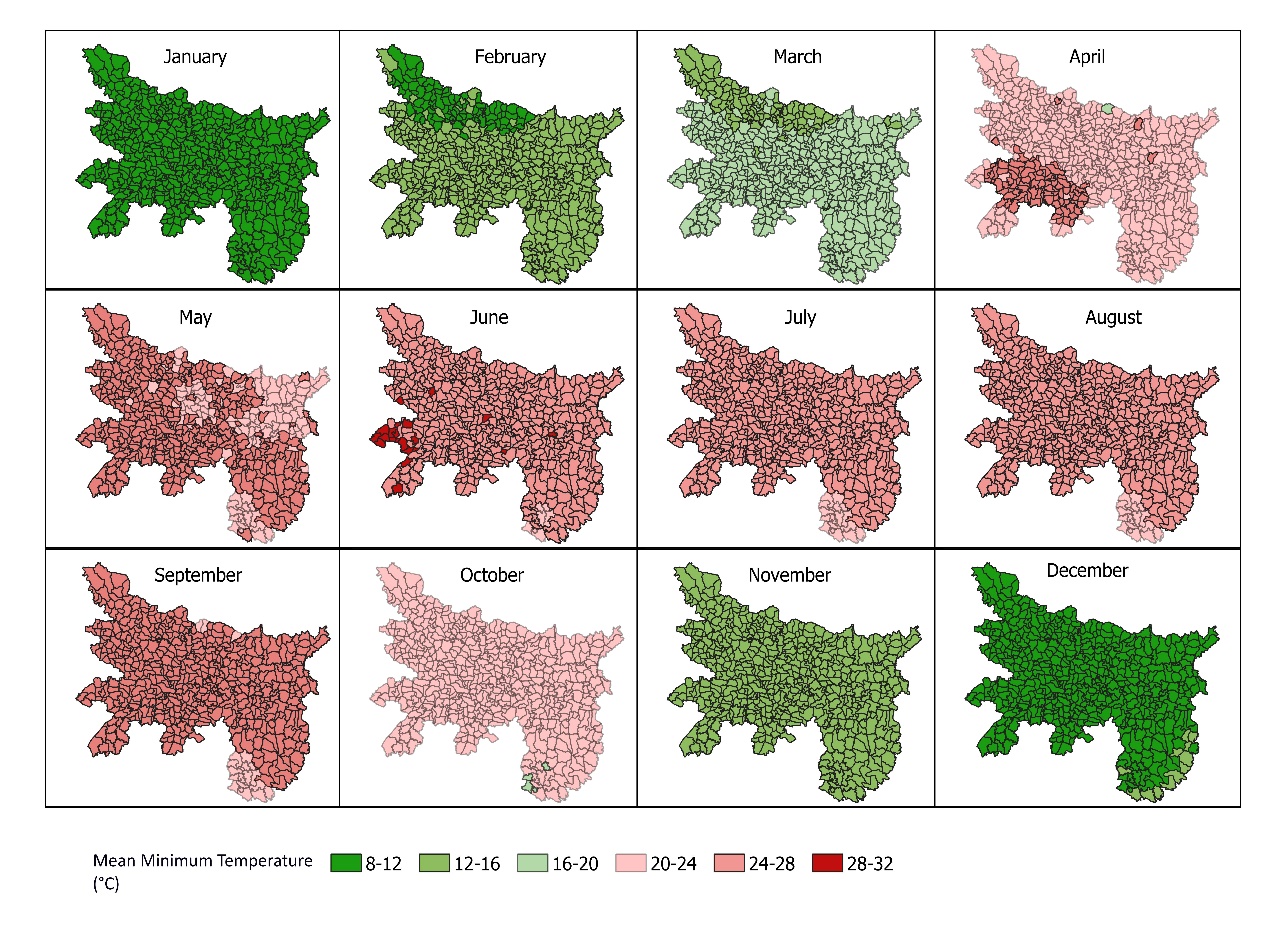
**Monthly minimum temperature (°C)**

1.
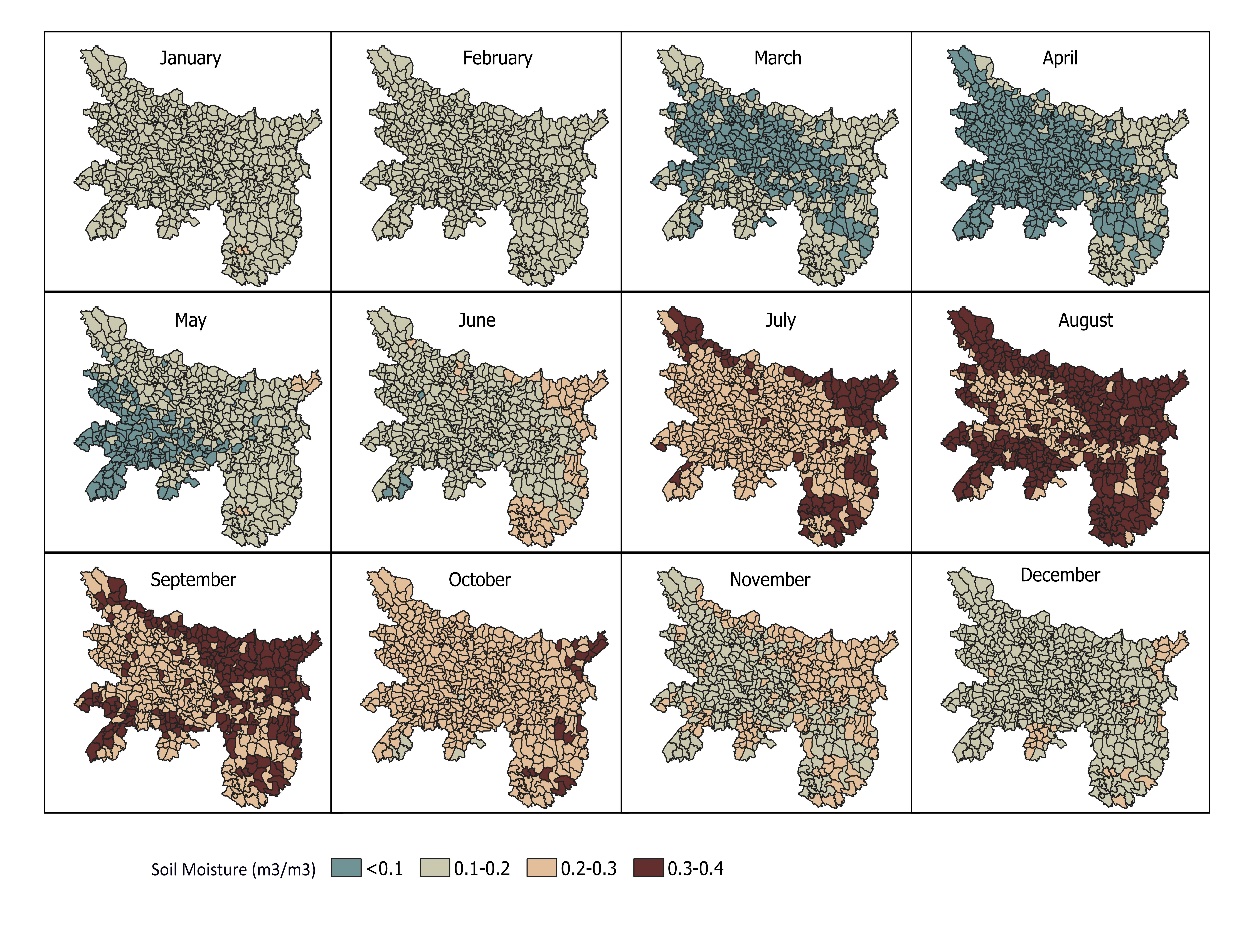
**Soil moisture (m^3^/m^3^)**

**(G) Population density per Km^2^**


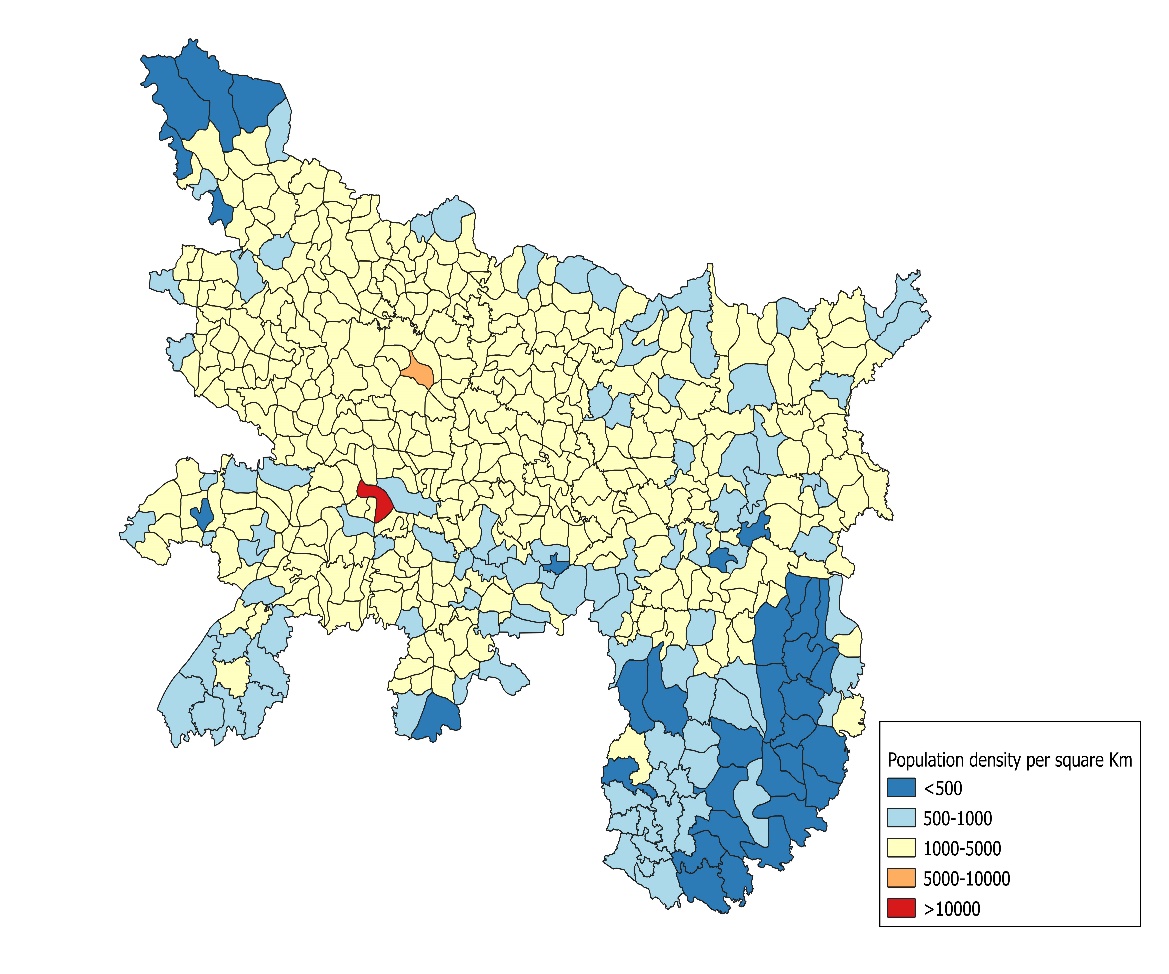


(H) Enhanced vegetation index (spectral index)
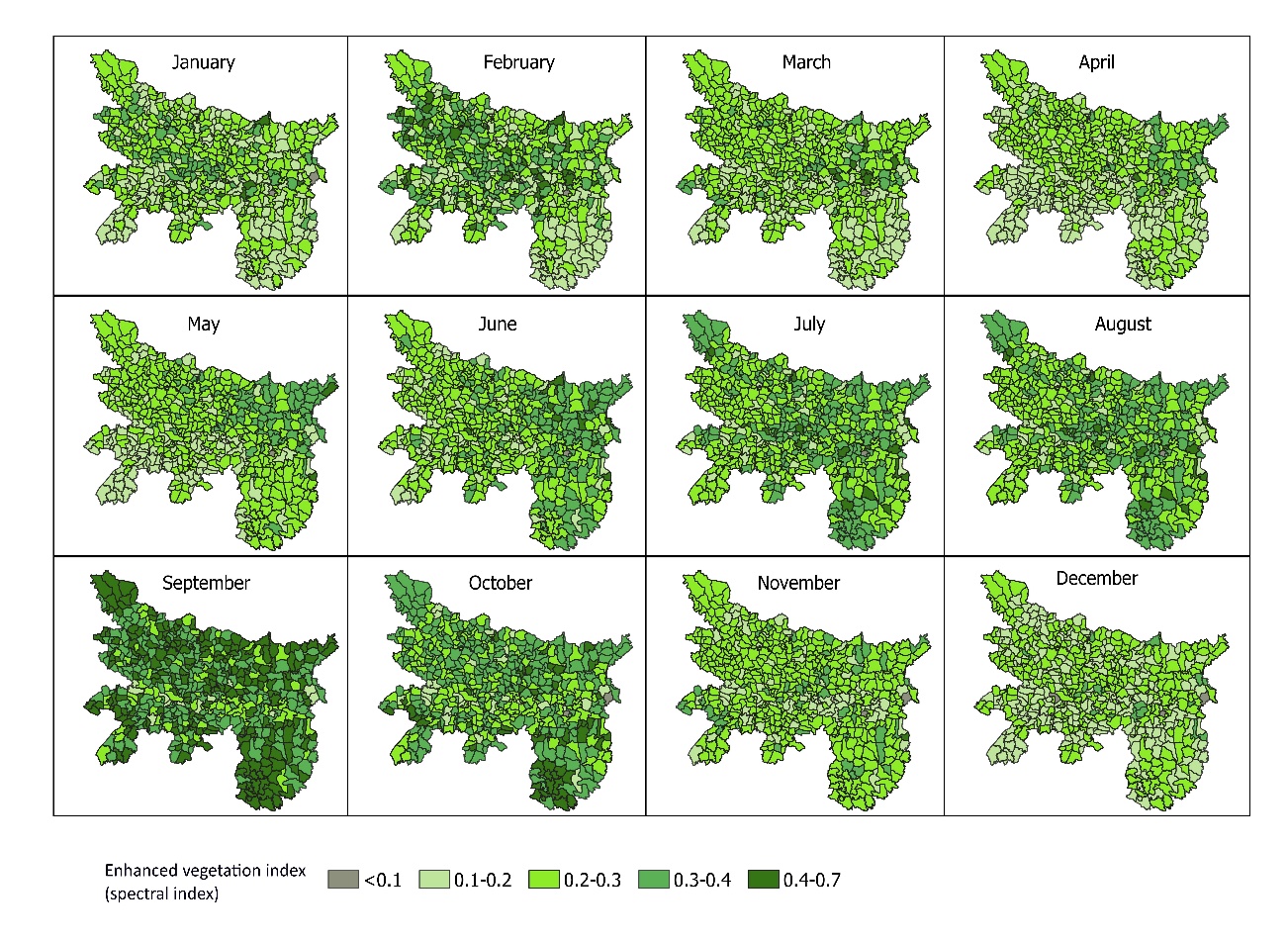


**(I) Land Surface Temperature (°C)**


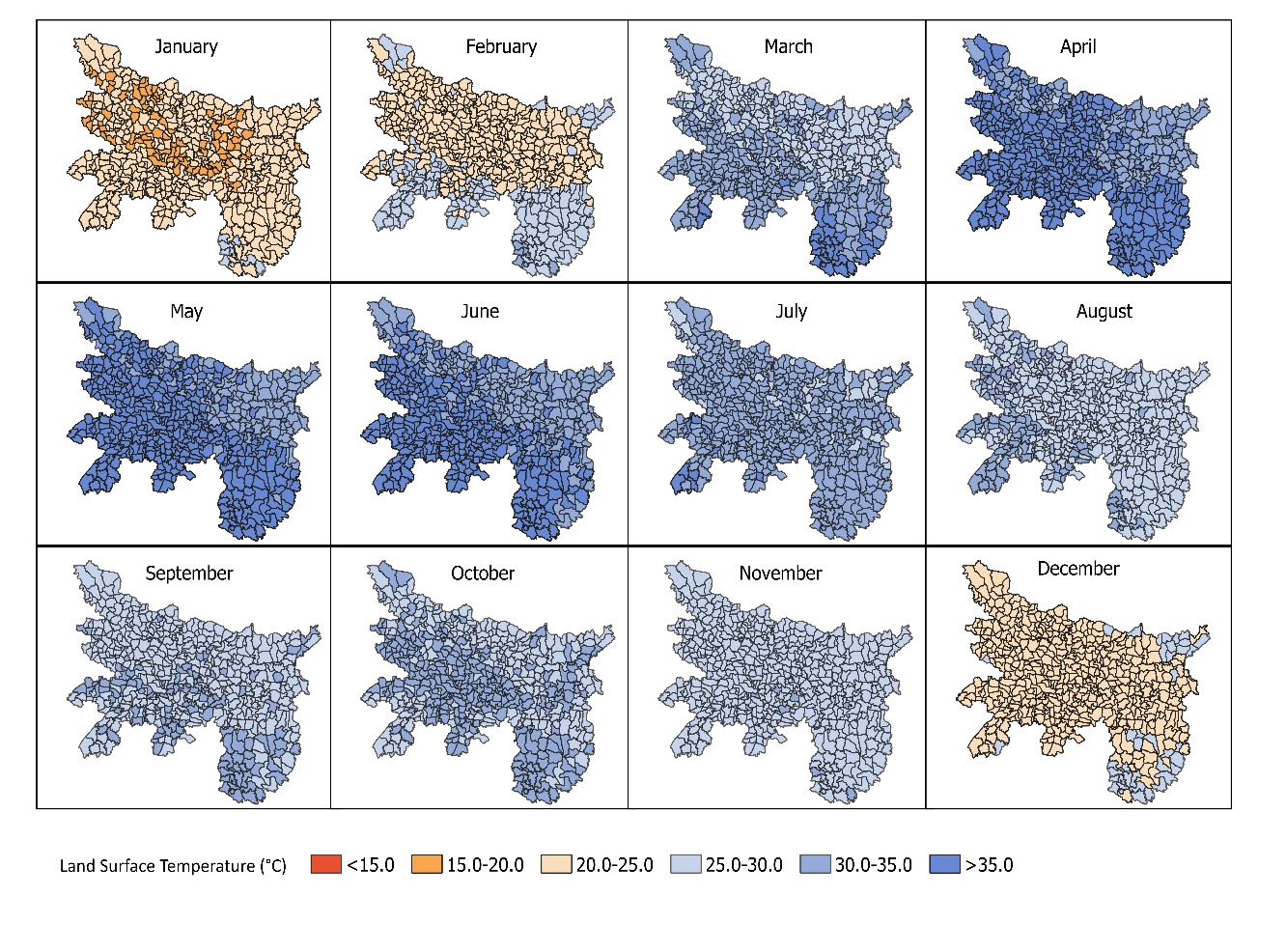

Supplement: S1 Fig — (A) Monthly mean temperature per month (BIO1,°C), (B) Isothermality (BIO3, %), (C) average precipitation per month (BIO12, mm), (D) Monthly maximum temperature (°C), (E) Monthly minimum temperature (°C), (F) Soil moisture (m3/m3), (G) Population density (per Km2), (H) Enhanced vegetation index (spectral index), (I) Land Surface Temperature (°C). Block level shapefile for Bihar and Jharkhand were developed in ArcGIS software (https://www.arcgis.com) by digitization tool using base layer from the India village directory, Census of India 2011, download from https://lgdirectory.gov.in. (DOCX) [file pntd.0011946.s001.docx]
