# Supplementary figures and images for: Modelling spatiotemporal patterns of visceral leishmaniasis incidence in two endemic states in India using environment, bioclimatic and demographic data, 2013–2022
Source: PLoS Negl Trop Dis. 2024 Feb 5;18(2):e0011946. doi: 10.1371/journal.pntd.0011946 (PMC10868833; doi:10.1371/journal.pntd.0011946)

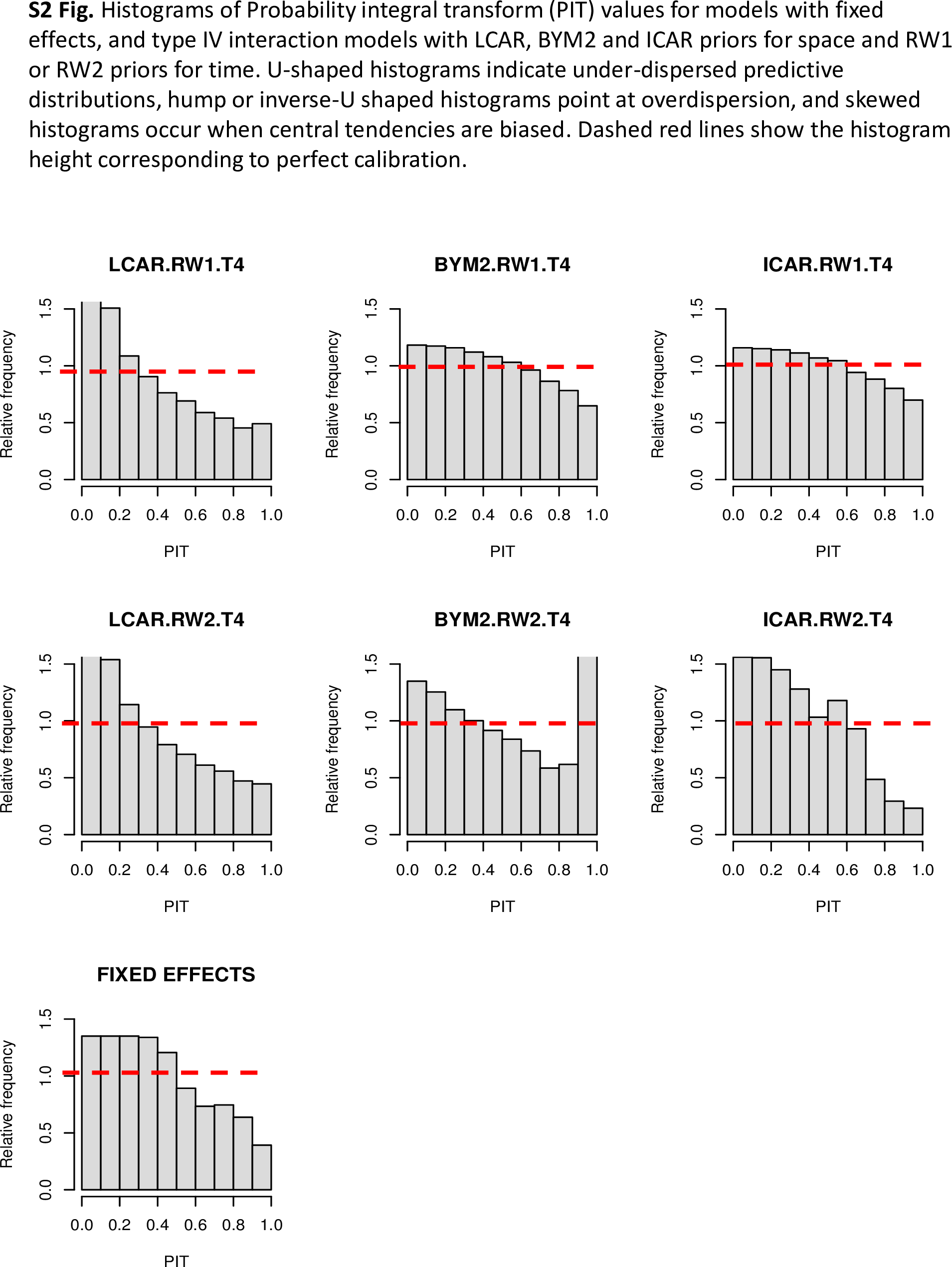

Supplement: S2 Fig — U shaped histograms indicate under-dispersed predictive distributions, hump or inverse-U shaped histograms point at overdispersion, and skewed histograms occur when central tendencies are biased. Dashed red lines show the histogram height corresponding to perfect calibration. (TIF) [file pntd.0011946.s002.tif]
