# Supplementary material for: Modelling spatiotemporal patterns of visceral leishmaniasis incidence in two endemic states in India using environment, bioclimatic and demographic data, 2013–2022
Source: PLoS Negl Trop Dis. 2024 Feb 5;18(2):e0011946. doi: 10.1371/journal.pntd.0011946 (PMC10868833; doi:10.1371/journal.pntd.0011946)
Supplement: S1 Table — (DOCX) [file pntd.0011946.s003.docx]

**S1 Table. Data source and properties of environment, bioclimatic and demographic variables**

| **S. No.** | **Data type** | **Data period**** | **Temporal resolution** | **Spatial resolution** | **Source** |
| --- | --- | --- | --- | --- | --- |
| 1 | Mean temperature (Bio 1, X_1_) | 2013 - 2023 | Monthly | ~1 km | https://worldclim.org |
| 2 | Isothermality (BIO 3, X_2_) ^#^ | 2013 - 2023 | Monthly | ~1 km | https://worldclim.org |
| 3 | Precipitation (BIO 12, X_3_) | 2013 - 2023 | Monthly | ~1 km | https://worldclim.org |
| 4 | Maximum temperature (X_4_) | 2013 - 2023 | Monthly | ~1 km | https://worldclim.org |
| 5 | Minimum temperature (X_5_) | 2013 - 2023 | Monthly | ~1 km | https://worldclim.org |
| 6 | Soil moisture (X_6_) | 2013 - 2023 | Monthly | 0.25° x 0.25° | Copernicus Dorigo, W., Scanlon, T., Reimer, C.; Van der Schalie, R.; Preimesberger, W., De Jeu, R. (2017): Soil moisture gridded data from 1978 to present, v201706.0.0. Copernicus Climate Change Service (C3S) Climate Data Store (CDS). |
| 7 | Population density (X_7_) * | 2013 - 2023 | 2011 Census | Block | Office of the Registrar General and Census Commissioner, India. http://censusindia.gov.in) |
| 8 | Enhanced vegetation index (X_8_) | 2013 - 2023 | Monthly | ~1 km | MODIS |
| 9 | Land surface temperature (X_9_) | 2013 - 2023 | Monthly | ~6 Km | MODIS |

* Monthly population for each block was projected forwards from Jan 2013 to Dec 2022 based on decadal growth rate of 2011 Census

citations

** Till March 2023

^#^Isothermality is calculated by the ratio of the mean diurnal range (difference between minimum and maximum daily temperature) to the annual temperature range.
