# Supplementary material for: Modelling spatiotemporal patterns of visceral leishmaniasis incidence in two endemic states in India using environment, bioclimatic and demographic data, 2013–2022
Source: PLoS Negl Trop Dis. 2024 Feb 5;18(2):e0011946. doi: 10.1371/journal.pntd.0011946 (PMC10868833; doi:10.1371/journal.pntd.0011946)
Supplement: S2 Table — (DOCX) [file pntd.0011946.s004.docx]

**S2 Table. Model selection criteria (DIC, P_D_ and WAIC) for the complete set of models**

| Model No. | Model | **Models with covariates** | | | | |
| --- | --- | --- | --- | --- | --- | --- |
|  |  | Spatial | Temporal | DIC | P_D_ | WAIC |
|  | **Additive models** | | | | | |
| 1 | Fixed effects (FE) | NA | NA | 103428.99 | 8.98 | 103429.85 |
| 2 | LCAR.IID.AD | LCAR | IID | 79060.96 | 560.76 | 79063.18 |
| 3 | LCAR.RW1.AD | LCAR | RW1 | 79058.30 | 549.73 | 79060.47 |
| 4 | LCAR.RW2.AD | LCAR | RW2 | 79108.63 | 560.85 | 79094.97 |
| 5 | BYM2.IID.AD | BYM2 | IID | 78996.91 | 519.01 | 79022.89 |
| 6 | BYM2.RW1.AD | BYM2 | RW1 | 78991.76 | 508.00 | 79017.58 |
| 7 | BYM2.RW2.AD | BYM2 | RW2 | 79047.98 | 493.06 | 79068.77 |
| 8 | ICAR.IID.AD | ICAR | IID | 78996.84 | 517.79 | 79023.67 |
| 9 | ICAR.RW1.AD | ICAR | RW1 | 78991.30 | 505.67 | 79018.07 |
| 10 | ICAR.RW2.AD | ICAR | RW2 | 79087.58 | 484.75 | 79108.62 |
|  | **Type I Interaction** | | | | | |
| 11 | LCAR.IID.T1 | LCAR | IID | 79072.92 | 1924.66 | 79058.91 |
| 12 | LCAR.RW1.T1 | LCAR | RW1 | 79065.44 | 1939.70 | 79051.78 |
| 13 | LCAR.RW2.T1 | LCAR | RW2 | 79065.06 | 1952.13 | 79051.49 |
| 14 | BYM2.IID.T1 | BYM2 | IID | 79079.44 | 1866.43 | 79067.20 |
| 15 | BYM2.RW1.T1 | BYM2 | RW1 | 79072.73 | 1887.09 | 79060.34 |
| 16 | BYM2.RW2.T1 | BYM2 | RW2 | 79116.64 | 1896.17 | 79104.14 |
| 17 | ICAR.IID.T1 | ICAR | IID | 78813.73 | 1835.62 | 83374.80 |
| 18 | ICAR.RW1.T1 | ICAR | RW1 | 78797.63 | 1861.77 | 83359.66 |
| 19 | ICAR.RW2.T1 | ICAR | RW2 | 78799.90 | 1847.63 | 83361.99 |
|  | **Type II interaction** | | | | | |
| 20 | LCAR.RW1.T2 | LCAR | RW1 | 75625.83 | 2492.96 | 75633.04 |
| 21 | LCAR.RW2.T2 | LCAR | RW2 | 78102.65 | 2614.72 | 95331.15 |
| 22 | BYM2.RW1.T2 | BYM2 | RW1 | 75603.45 | 2436.31 | 75634.33 |
| 23 | BYM2.RW2.T2 | BYM2 | RW2 | 78129.04 | 2611.86 | 96457.73 |
| 24 | ICAR.RW1.T2 | ICAR | RW1 | 75584.04 | 2405.90 | 75640.21 |
| 25 | ICAR.RW2.T2 | ICAR | RW2 | 81968.05 | 4298.00 | 193514.53 |
|  | **Type III interaction** | | | | | |
| 26 | LCAR.RW1.T3 | LCAR | RW1 | 77478.51 | 2950.91 | 77541.42 |
| 27 | LCAR.RW2.T3 | LCAR | RW2 | 77491.60 | 2900.31 | 77558.14 |
| 28 | BYM2.RW1.T3 | BYM2 | RW1 | 77527.57 | 2869.01 | 77586.83 |
| 29 | BYM2.RW2.T3 | BYM2 | RW2 | 77557.47 | 2919.66 | 77601.71 |
| 30 | ICAR.RW1.T3 | ICAR | RW1 | 77517.66 | 2959.51 | 77566.78 |
| 31 | ICAR.RW2.T3 | ICAR | RW2 | 77531.16 | 2946.02 | 77580.91 |
|  | **Type IV interaction** | | | | | |
| 32 | LCAR.RW1.T4 | LCAR | RW1 | 75310.43 | 2288.36 | 75322.46 |
| 33 | LCAR.RW2.T4 | LCAR | RW2 | 78245.24 | 2782.26 | 113141.22 |
| **34** | **BYM2.RW1.T4** | **BYM2** | **RW1** | **75175.76** | **2231.99** | **75256.90** |
| 35 | BYM2.RW2.T4 | BYM2 | RW2 | 79147.32 | 3151.72 | 119035.32 |
| **36** | **ICAR.RW1.T4** | **ICAR** | **RW1** | **75159.58** | **2216.24** | **75252.72** |
| 37 | ICAR.RW2.T4 | ICAR | RW2 | 79175.41 | 3152.72 | 119977.92 |
| AD | Additive model | | | | | |
| IID | Independent Identically Distributed model for temporal random effect | | | | | |
| LCAR | Leroux Conditional Auto-Regressive model for spatial random effect | | | | | |
| BYM2 | Besag-York-Mollie model for spatial random effect | | | | | |
| ICAR | Intrinsic Conditional Auto-Regressive model for spatial random effect | | | | | |
| RWx | Random walk model of order ‘x’ for temporal random effect | | | | | |
| Tx | Type ‘x’ of space-time interaction model | | | | | |
| Best fitting models (34 & 36) are highlighted in red | | | | | | |
