# Supplementary material for: Modelling spatiotemporal patterns of visceral leishmaniasis incidence in two endemic states in India using environment, bioclimatic and demographic data, 2013–2022
Source: PLoS Negl Trop Dis. 2024 Feb 5;18(2):e0011946. doi: 10.1371/journal.pntd.0011946 (PMC10868833; doi:10.1371/journal.pntd.0011946)
Supplement: S3 Table — (DOCX) [file pntd.0011946.s005.docx]

**S3 Table List of blocks with annual VL incidence above the elimination threshold (1/ 10,000 population) in the states of Bihar and Jharkhand during 2021-2023**

| **State** | **District** | **Block** | **Predicted annual incidence per 10,000 population by year** | | |
| --- | --- | --- | --- | --- | --- |
|  |  |  | **2021** | **2022** | **2023** |
| **BIHAR** | ARARIA | RANIGANJ_A | **0.90** | **0.98** | 1.01 |
| **BIHAR** | GOPALGANJ | BARAULI | 1.58 | 1.80 | 1.72 |
| **BIHAR** | GOPALGANJ | BHOREY | 1.12 | 1.28 | 1.25 |
| **BIHAR** | GOPALGANJ | GOPALGANJ | **0.96** | 1.12 | 1.08 |
| **BIHAR** | GOPALGANJ | MANJHA | 1.75 | 1.98 | 1.88 |
| **BIHAR** | GOPALGANJ | PACH DEURI | 1.11 | 1.27 | 1.24 |
| **BIHAR** | GOPALGANJ | SIDHWALIA | 1.43 | 1.63 | 1.57 |
| **BIHAR** | KISHANGANJ | TERHAGACHH | 1.12 | 1.25 | 1.37 |
| **BIHAR** | MUZAFFARPUR | BOCHAHA | **0.91** | 1.06 | 1.03 |
| **BIHAR** | MUZAFFARPUR | PAROO | 1.76 | 1.99 | 1.88 |
| **BIHAR** | MUZAFFARPUR | SAHEBGANJ | 1.41 | 1.61 | 1.53 |
| **BIHAR** | NAWADA | KASHI CHAK | 1.47 | 1.69 | 1.68 |
| **BIHAR** | PURBA CHAMPARAN | TETARIA | 1.18 | 1.35 | 1.29 |
| **BIHAR** | PURNIA | JALALGARH | **1.00** | 1.06 | 1.10 |
| **BIHAR** | SARAN | AMNOUR | 1.91 | 2.14 | 2.05 |
| **BIHAR** | SARAN | BANIAPUR | 1.39 | 1.54 | 1.46 |
| **BIHAR** | SARAN | DARIAPUR | **2.26** | 2.45 | 2.32 |
| **BIHAR** | SARAN | GARKHA | 2.88 | 3.15 | 2.98 |
| **BIHAR** | SARAN | ISHUPUR | 1.72 | 1.96 | 1.87 |
| **BIHAR** | SARAN | LAHLADPUR | 3.36 | 3.81 | 3.63 |
| **BIHAR** | SARAN | MAKER | 1.94 | 2.22 | 2.13 |
| **BIHAR** | SARAN | MARHAURA | 1.77 | 1.97 | 1.89 |
| **BIHAR** | SARAN | MASHRAKH | 1.33 | 1.52 | 1.46 |
| **BIHAR** | SARAN | NAGRA | 2.44 | 2.71 | 2.55 |
| **BIHAR** | SARAN | PANAPUR | **0.99** | 1.19 | 1.17 |
| **BIHAR** | SARAN | PARSA | 3.58 | 3.96 | 3.78 |
| **BIHAR** | SARAN | REVELGANJ | 1.03 | 1.20 | 1.16 |
| **BIHAR** | SARAN | SONEPUR | 2.40 | 2.70 | 2.54 |
| **BIHAR** | SARAN | TARAIYA | 1.62 | 1.83 | 1.76 |
| **BIHAR** | SIWAN | BARHARIA | 1.21 | 1.40 | 1.35 |
| **BIHAR** | SIWAN | BASANTPUR_S | 1.89 | 2.14 | 2.05 |
| **BIHAR** | SIWAN | BHAGWANPUR HAT | 1.52 | 1.73 | 1.69 |
| **BIHAR** | SIWAN | GORIAKOTHI | 3.06 | 3.39 | 3.20 |
| **BIHAR** | SIWAN | LAKRI NABIGANJ | 1.79 | 2.05 | 1.97 |
| **BIHAR** | SIWAN | MAHARAJGANJ_S | 1.11 | 1.29 | 1.25 |
| **BIHAR** | SIWAN | NAUTAN | 1.07 | 1.22 | 1.18 |
| **BIHAR** | SIWAN | PACHRUKHI | 1.23 | 1.41 | 1.38 |
| **BIHAR** | VAISHALI | RAGHOPUR | 1.44 | 1.66 | 1.60 |
| **Total no. of blocks above elimination threshold** | | | **33** | **37** | **38** |
| **JHARKHAND** | DUMKA | GOPIKANDAR | 1.84 | 1.99 | 1.94 |
| **JHARKHAND** | DUMKA | JAMA | 2.74 | 2.98 | 2.99 |
| **JHARKHAND** | DUMKA | KATHIKUND | 4.03 | 4.34 | 4.34 |
| **JHARKHAND** | DUMKA | RAMGARH_D | 1.95 | 2.17 | 2.17 |
| **JHARKHAND** | DUMKA | SHIKARIPARA | **0.88** | **0.99** | 1.01 |
| **JHARKHAND** | GODDA | BOARIJOR | 4.02 | 4.33 | 4.35 |
| **JHARKHAND** | GODDA | POREYAHAT | **1.00** | 1.11 | 1.13 |
| **JHARKHAND** | GODDA | SUNDARPAHARI | 2.64 | 2.88 | 2.89 |
| **JHARKHAND** | PAKAUR | AMRAPARA | 4.91 | 5.28 | 5.27 |
| **JHARKHAND** | PAKAUR | HIRANPUR | 3.07 | 3.29 | 3.35 |
| **JHARKHAND** | PAKAUR | LITIPARA | 2.38 | 2.58 | 2.67 |
| **JHARKHAND** | PAKAUR | MAHESHPUR | 2.01 | 2.20 | 2.20 |
| **JHARKHAND** | PAKAUR | PAKAUR | 1.96 | 2.14 | 2.12 |
| **JHARKHAND** | SAHIBGANJ | BARHAIT | 1.47 | 1.61 | 1.68 |
| **JHARKHAND** | SAHIBGANJ | BORIO | 2.03 | 2.21 | 2.28 |
| **JHARKHAND** | SAHIBGANJ | MANDRO | 2.24 | 2.46 | 2.52 |
| **JHARKHAND** | SAHIBGANJ | PATHNA | 1.86 | 2.06 | 2.12 |
| **JHARKHAND** | SAHIBGANJ | TALJHARI | 1.41 | 1.56 | 1.60 |
| **Total no. of blocks above elimination threshold** | | | **16** | **17** | **18** |
| **Total no. of blocks above elimination threshold (BIHAR + JHARKHAND)** | | | **49** | **54** | **56** |

***Blocks in which incidence is above the elimination threshold are colored in red***
