# Supplementary material for: Modelling spatiotemporal patterns of visceral leishmaniasis incidence in two endemic states in India using environment, bioclimatic and demographic data, 2013–2022
Source: PLoS Negl Trop Dis. 2024 Feb 5;18(2):e0011946. doi: 10.1371/journal.pntd.0011946 (PMC10868833; doi:10.1371/journal.pntd.0011946)
Supplement: Attachment — Submitted filename: response to Reviewers comments.docx [file pntd.0011946.s006.docx]

The Editors,

PLoS NTDs

07 January 2024

# **Ref:** Modelling spatiotemporal patterns of visceral leishmaniasis incidence in India using environment, bioclimatic and demographic data, 2013-2021 **(PNTD-D-23-00561)**

Dear Editors,

We have uploaded two versions of the revised manuscript: one with tracked changes denoting where the text has been changed and the other a clean version.

All the figures, Tables and Supplementary information were uploaded after removing all technical errors pointed out by the PLOS NTD Team.

Also, uploaded a detailed list of responses to the reviewers comments and a description of the changes made in the revised manuscript.

We have changed the manuscript significantly to address the reviewers’ comments and believe that it has made a substantial improvement.

We would like to thank the reviewers for their constructive comments / suggestions on our manuscript, which helped us to improve the manuscript substantially.

Thank you for your patience and support in agreeing to our requests for extending the due date for submission. We hope the revised ms will meet the journal requirement and look forward to receiving editorial decision.

Kind regards, also on behalf of all authors

Swaminathan Subramanian

===============================

**METHODS**

**Reviewer #1:**

**I am not a modeler but I assume methods are correct**

***Response:*** *We are grateful to the reviewer for his other critical major comments, which are really thought provoking and helped us while revising the ms.*

**Reviewer #2:**

**It is necessary to provide a more detailed explanation of how the blocks, which served as the units of analysis are constructed. How big are they? Is there a lot of variability? Why were blocks chosen as the units of analysis?**

***Response:*** *Thank you for the thoughtful comment. A block is a second level administrative unit after ‘district’. A district may consist of several blocks, the next level of administrative units. Mostly, the blocks match with the health units of a district, the Community Health Centres. In Bihar, the area of the blocks varies between 11 and 835 sq. kms (mean: 168 sq. km), and the population range between 31,509 and 18,22,776 (mean: 207,969; 2013 projected population from 2011 census data). The population density varies between 199 and 10238 per sq. km (mean: 1410.5).*

*In Jharkhand, the area varies between 86.5 and 516.1 sq. km (mean:298.4 sq. km) and the population between 43,443 and 3,50,129 (mean: 1,47,982, projected from 2011 census data) in the 33 endemic blocks. The population density varies between 154 and 1770 per sq. km (mean: 602).*

*The blocks were chosen as the unit of analysis as the national programme aims to achieve VL elimination at block level, the cut-off incidence being defined as 1 case per 10,000 population per year per block.*

*In the revised ms, we have incorporated the above descriptions by adding a section on ‘Study area’ with a map showing the endemic and non-endemic blocks included in the study* ***(Please see pg8-10, LL181-234).***

**To enhance the description of the study area, I recommend including a map of India highlighting the specific regions under investigation.**

***Response:*** *As suggested, we have included a map of India (Fig 1) highlighting the specific regions under investigation (458 endemic blocks and 11 non-endemic blocks in Bihar state and 33 endemic and 16 non-endemic blocks in Jharkhand state).*

**Furthermore, it is essential to provide better explanation of how the priors were defined. Clarifying the process will help readers comprehend the underlying assumptions and establish a stronger foundation for the subsequent analyses.**

***Response:*** *As suggested, a detailed description of the various priors used to model the spatial, temporal, and spatiotemporal random effects are incorporated in the revised ms* ***(Please see pg16-17, LL365-396).***

**Authors mention that restricted models or orthogonal analysis were not conducted due to computational constraints. It would be beneficial to explicitly outline these constraints, enabling readers to better evaluate the feasibility of such analyses. If conducting these analyses is genuinely impossible, please provide a thorough justification for this. On the contrary, such analyzes must be carried out.**

***Response:*** *We tried to apply the restricted regression methodology to alleviate the confounding effects of spatiotemporal interaction models using a Windows (ver 10) based desktop computer with 256 GB RAM memory. While carrying out the above analysis, the system halted after a week of running with an error of “memory exhausted”. We understand that with type IV interaction models, when the number of locations and/or the number of time points is large (data (518 blocks x 10 years x 12 months per year=61440 records), the inference gets computationally challenging due to the high number of required constraints necessary for inference with type IV interaction models (Esmail et al., 2022, Approximate Bayesian Inference for the Interaction Types 1, 2, 3 and 4 with Application in Disease Mapping, a preprint in Research Gate).*

*Adin et al, (2023) developed methods to alleviate confounding effects in spatiotemporal areal models with application on crime against women in India (Statistical Modelling, doi:* [*https://doi.org/10.1177/1471082X211015452*](https://doi.org/10.1177/1471082X211015452)*). These authors reported that fitting a classical spatiotemporal random effect model (type IV interaction model) led to a good fit of the spatiotemporal patterns of risk, but they fail to account for the correct linear association between the response and the covariates. A model with only covariates (fixed effects model) provides unbiased estimates of the fixed effects coefficients but it gives a poor fit as it does not capture the variability unexplained by the covariates. The restricted regression considered by Adin et al., offers in one single model the best of the two approaches: similar to the model without random effects (fixed effects model), it provides correct estimates of the fixed effects and prediction of spatiotemporal patterns of risk. This is an advantage of the restricted regression approach over the fixed effect model (without random effects) and classical spatiotemporal models with type IV interaction. These authors, however, concluded that ignoring confounding effects, based on classical type IV interaction models with covariates, will not be a problem if the primary interest is estimating relative risks and predicting the outcomes.*

*Since the fixed effects estimates of the covariates are proven to be comparable between a model with covariates alone and type IV interaction model with covariates based on restricted regression or orthogonality constraints approach, we measured the association of the covariates with VL incidence based on fixed effects model, and the classical type IV interaction model for predicting, and forecasting VL incidence at block level. We have incorporated the above explanations in the revised ms* ***(Pl. see pg33-34, LL755-771 & pg36-37, LL833-842).***

**Reviewer #3:**

The objectives are consistent with the stated hypotheses.
The study design is appropriate and well detailed. It is a complementary study, adds temporal data and evaluation of variables that improve the results and applications on a larger scale.
The ethical standards are described and complied with.
Add a general map where Bihar and Jharkhand are geographically located.

***Response:*** *Thanks for your appreciation and suggestion. We have added a map showing the study area and its geographical location in India (****Please see Figure 1).***

--------------------

**Results**

-Does the analysis presented match the analysis plan?
-Are the results clearly and completely presented?
-Are the figures (Tables, Images) of sufficient quality for clarity?

**Reviewer #1:** Yes

***Response:*** *Thanks*

**Reviewer #2:**

**In Table 1, it is observed that in 2020, some variables exhibit correlation coefficients that significantly deviate from those of previous years. It would be valuable to explain and discuss the reasons behind this discrepancy in detail.**

**Response:** In 2020, variation in correlation coefficients for some variables could have been disturbed by the varied levels of interventions, including surveillance and treatment, as a result of COVID-19 pandemic.

In the revised ms, we have re-analyzed the data separately for space (518 blocks) and time (8 years, 2013-2020) to understand the spatial and temporal patterns of correlations between each covariate and VL incidence. For each block, the monthly mean value of each covariate was obtained by taking average for all the years from 2013-2020 and was related to overall monthly VL incidence. The Pearson correlation coefficients for all the 518 blocks were plotted against each covariate in a box-plot to show the spatial variation in association. Similarly, year-wise Pearson correlation coefficient was obtained by relating monthly mean value of for each covariate with monthly overall VL incidence from all the 518 blocks. The year-wise correlation was plotted against each covariate to show the temporal variation in their association with incidence. The spatial and temporal patterns of correlations for each covariate was examined for their significance conservatively at P<0.2. Covariates with a P value less than 0.2 in at least one of the years (2013-2020) or one of the 518 blocks were subsequently included in the spatiotemporal analysis. The association of each covariate was then inferred after fitting a model with covariates alone (fixed effects) and spatiotemporal models with covariates **(Please see pg 20-22, LL472-501; pg24, LL560-567; Table1)**

**In Figure 1, please include a legend for the grey color used in the visual representation to ensure readers can accurately interpret the information presented.**

***Response:*** *Figure legend is updated adding legend to grey colour, and also for the coloured lines for better interpretation.*

**Reviewer #3: The results are presented in a clear and orderly manner; figures, tables and supplementary material are sufficient.**

***Response:*** *Thanks*

--------------------

**Conclusions**

-Are the conclusions supported by the data presented?
-Are the limitations of analysis clearly described?
-Do the authors discuss how these data can be helpful to advance our understanding of the topic under study? -Is public health relevance addressed?

**Reviewer #1:**

See summary and general comments

***Response:*** *Please see response in Summary and General comments*

**Reviewer #2:**

**The results regarding population density require a more in-depth discussion. Why is the incidence higher in less dense areas? Why is this the only significant variable in the models?** **Why did the other variables lose their significance? Although you briefly mention these points in the limitations section, I recommend further elaboration.**

***Response:***

*The negative correlation in our study could be attributed to the fact that a large proportion of population at risk (>75%) live in rural blocks with low population density (Bihar: 1005.4 in rural and 5057.8 per km^2^ in urban; Jharkhand: 323.4 in rural and 3527.5 per km^2^ in urban) and abundant vegetation (as indicated by EVI), which is positively associated with VL incidence. In India. Kesari et al. [58] reported that NDVI was positively associated with VL incidence. In Brazil it was found that areas with high population density and abundant vegetation are associated with high VL incidence [reference 64 cited in the revised ms]. Therefore, abundance of vegetation seems to make the difference. Sandfly density (human-vector contact) is expected to be higher in rural areas, where population density is low, vegetation abundant, and sandfly breeding habitats are highly prevalent.*

*Please see response to a comment of Reviewer 3 below, wherein we have explained the reasons for ‘population density’ is the only significant variable in the best model (type IV interaction with covariates).*

*Also, as suggested by Reviewer 1, we have now included 16 non-endemic blocks bordering endemic blocks in the state of Jharkhand, updated covariates data for the period 2021-2022, and refitted all the 37 models with covariates. The results showed that while all the covariates were significantly associated positively or negatively with VL incidence based on a model covariate alone, only population density and LST are negatively correlated with VL incidence for the best fitting type IV interaction model with covariates. The negative association of latter two variables are in the same direction as in covariates only model. However, we used the results of fixed effect model to assess the associations of covariates with VL incidence (pl. also see response to reviewer 3 under “Conclusions” for more explanation). We have incorporated the above result and elaborated in the Discussion under limitation* ***(Please see pg32-33, L729-749 & pg33-34, 755-771).***

**It is crucial to include a discussion on data quality and whether potential biases could have influenced the study's findings.**

***Response:*** *We have added a para in the revised ms under Discussion on data quality related to change in surveillance activity, diagnostic accuracy over time due coinfections (VL with HIV or COVID 19) and potential biases that could have influenced our model predictions. We have discussed about quality and the potential bias that could have influenced the study findings* ***(please see pg36, LL821-831)***

**Conclusions should offer more objective insights regarding the implications of the results for health interventions. Consider providing more explicit recommendations or directions for future actions based on the findings of your study.**

***Response:*** *We have added the implications of the study and recommendations in the “Conclusion” section of the revised ms* ***(Pl. see pg37-38, LL858-871).***

**Reviewer #3:**

**There are no limitations beyond finding a single endemic factor, probably further investigation can be done with variables related to "urbanisation" or the possibility of including vector abundance from available dynamics studies. In relation to the likely biases in VL case reports, the COVID pandemic is cited as a drawback.**

***Response:*** *We agree with reviewer’s suggestion to carry out further investigation with variable related to “urbanization” and “vector abundance”. However, a closer look at the block level population data reveals that >75% of the population live in rural areas both in the states of Bihar and Jhrakhand* ***(please see Section on “Study area” in the Revised ms).*** *The role of urbanization seems to be minimal.*

*It could have certainly been better to include data on vector abundance but unfortunately, such data are not available for all the blocks for the entire study period (Jan 2013-Dec 2022).*

*We have cited COVID pandemic as a limitation, apart from other bias, as the VL cases could have been missed during COVID period as fever is one of the major symptoms for both VL and COVID. However, we do not have data on whether all the fever cases were subjected to VL diagnosis or might have been considered as COVID cases due to gravity of COVID situations during that period* ***(Please see pg36, LL 821-831)****.*

--------------------

**Editorial and Data Presentation Modifications?**

Use this section for editorial suggestions as well as relatively minor modifications of existing data that would enhance clarity. If the only modifications needed are minor and/or editorial, you may wish to recommend “Minor Revision” or “Accept”.

**Reviewer #1:** (No Response)

**Reviewer #2:** (No Response)

**Reviewer #3:** (No Response)

——————————

**Summary and General Comments**

**Reviewer #1:**

Modelling spatiotemporal patterns of visceral leishmaniasis incidence in India using environment, bioclimatic and demographic data, 2013-2021

The authors have developed a model to predict incidence of VL at block level in Bihar and Jharkhand. They fitted data over the period 2013-2018 in a model incorporating environment, climatic and demographic factors. The model was validated with the monthly cases for 2019-2020, assessing the proportion of observations that fall inside the 95% credible interval for the predicted number of VL cases per month. During the training period model predictions agreed for > 93% with observed numbers of cases, over the testing period agreement went up to 99%. The authors conclude that the forecast can be used to monitor progress of VL elimination at the block level, to select blocks with incidence above the elimination threshold for targeted interventions and to prevent or reduce risk of resurgence in post- elimination settings.

**Major comments:**
This is a well-written manuscript but I would like to have some more clarity on the practical implications of using this model in the context of the VL elimination program in India. Outcomes are expressed in rates per 10,000 but what does that mean at block level? As numbers go further down, stochasticity will become more of an issue. Having 20 instead of 10 cases in a year at block level means a doubling of the rates but for program management purposes it may not be that much of a difference.

***Response:*** *We think having 20 instead of 10 cases in a year at block level do matter to the program as it crosses the elimination threshold.The elimination thresholds are defined at the block level. As the blocks are heterogeneous in terms of population size, the impact of a small outbreak in a block will depend on the size of the block (i.e. whether the number of cases exceeds the 1/10,000 threshold or not). We agree, given the small numbers of cases usually observed, and the large number of blocks (>600) stochastic effects will tend to dominate the patterns. In such situation, the focus should move down to village level in a block, rather than number of cases in the block overall. Our model forecasts VL incidence based on the probability of reaching elimination at block level. The model could be used as a tool to monitor trends in progress towards elimination and early signs of deviation in any particular block. This should trigger more targeted actions, intensified surveillance, treatment, and prevention focused measures at village level within a block* ***(Please see pg35, LL793-804).***

**When it comes to monitoring elimination and preventing resurgence, what does this model add to epidemiological surveillance? Either case-based surveillance or serological surveillance would provide more reliable information for monitoring transmission.**

***Response:*** *The results of recent xenodiagnoses studies in animals or humans in India (Singh et al, 2021) showed that sandfly infections in endemic areas are contributed primarily by patients with active visceral leishmaniasis or post-kala-azar dermal leishmaniasis (PKDL) and to a much lesser extent by treated patients and rarely or not at all by asymptomatic individuals. However, it is not clear how many of VL/PKDL cases are being missed during surveillance. These findings suggest that early case detection followed by complete treatment of cases could effectively remove these individuals as infection reservoirs. Such a strategy combined with vector control is reported to be sufficient to sustain the elimination effort. Our model, apart from monitoring elimination, can predict the risk of resurgence at the block level so that the program can be prepared in advance to take appropriate measures.*

*By including PKDL cases in the model, we can be more effective in predicting VL incidence and the risk of resurgence. We have elaborated the above points under discussion indicating the need for modelling PKDL cases in endemic blocks along with covariates, including vector density to gain more insight on the incidence of VL, especially when incidence in the blocks is maintained below the elimination threshold* ***(Please see pg35, LL806-812).***

**The authors selected 458 endemic blocks from Bihar as well as 11 non-endemic blocks and 33 endemic blocks from Jharkhand. How comparable were the non-endemic blocks from Bihar to endemic blocks? Were they rural blocks and where were they situated? And why were there no non-endemic blocks from Jharkhand included as in Jharkhand VL is much more limited in distribution. Would it not be interesting to see how the model performs when including non-endemic rural blocks of Jharkhand? This might be even more relevant in view of observations from Nepal where VL now pops up in areas where it never was before.**

***Response:*** *All the non-endemic blocks are rural and comparable with endemic blocks in terms of population density in both the states.*

*The administrative block units for urban and rural areas are classified using the Census of India 2011 criteria. The total number of non-endemic VL blocks in Bihar was 65, with 63 being rural and 2 being urban settlements. Similarly, in Jharkhand, the total number of non-endemic blocks was 227, with 207 being rural and 20 being urban settlements. The study includes 11 and 16 non-endemic blocks in Bihar and Jharkhand, respectively, with rural settlements.*

*As suggested, we have included 16 non-endemic blocks bordering the endemic blocks in Jharkhand for modelling and the results are presented in the revised ms* ***(Please see pg10, LL220-234).***

--------------------

**Minor comments:**
**In line 374-375 it is stated that: ‘Consequently, the blocks with low population density tend to have high incidence of VL and vice versa’. This seems intuitive to me because VL is a rural disease. But on page 26 (no more line numbers there) the authors state that: ‘Our findings on the positive association of population density with VL incidence supports the earlier work in Teresina, Piauí State, Brazil that areas with high population density and abundant vegetation are associated with high VL incidence [64]’. Is this not contradictory?**

***Response:*** *We have reanalyzed the data after extracting and including the data for the period 2021-2022 (as suggested by reviewer). We found that the association of population density with VL incidence is negative and significant. The results are comparable between the fixed effect and type IV interaction models* ***(pl. also see response to reviewer 3 under “Conclusions” for more explanation).***

**Data presented are until May, 2021. But we are two years later now and the KAMIS database is kept up to date. Would it not be possible to assess how well the predictions worked until the end of 2022 to get a stronger indication of how useful the model could be for forecasting VL incidence at block level?**

***Response:*** *Thanks for the suggestion: We have incorporated the covariates data until Dec 2022 and the best model was used to predict VL incidence until Dec 2023. For predicting VL incidence in 2023 we used monthly extracted covariates data from Jan 2023-Mar 2023 and the average values derived for each covariate based on the remotely sensed data extracted for the 10 years period, Apr 2013 - Dec 2022. However, we are unable to obtain the data from KAMIS database* ***(Please see pg12, LL269-271 & pg18, LL412-415).***

**Reviewer #2:**

**The study provides valuable insights into the incidence of visceral leishmaniasis in specific regions of India. The topic is highly relevant and contributes to our understanding of the factors influencing the spatial and temporal evolution of the disease.
However, I would like to offer some suggestions to improve the clarity and accessibility of your article. Other points that I consider relevant and essential for improving the study were described in the previous answers.**

**Firstly, the author summary could benefit from a clearer and more accessible language to ensure readers less familiar with the subject matter can easily comprehend the content. Additionally, several sections of the text require additional explanatory information to enhance understanding for readers who may be less familiar with the specific analyses performed.**

***Response:***

*Thanks for the comments. As the reviewer suggested, we have revised the text in the “Abstract” and incorporated additional explanatory information (priors used for modelling) for better understanding of the non-technical readers* ***(pl. see pg2-3, LL34-52 and pg16-17, LL365-396****)*

**Regarding the title, it would be beneficial to specify that the study focuses solely on two states of India, providing a more concise and accurate representation of the research scope.**

***Response:*** *Done. Title revised as “Modelling spatiotemporal patterns of visceral leishmaniasis incidence in two endemic states in India using environment, bioclimatic and demographic data, 2013-2022”.*

**Reviewer #3:**

**The problem of spatial and temporal scale is a well-known and much-discussed issue when investigating an early warning and response system in the generation of leishmaniasis outbreaks. In general, phlebotomine sandfly species tend to be microfocal and it is imperative to have possible interventions at different spatial-temporal scales, which makes this type of approximation studies extremely essential to apply in public health in order to know the system of the area studied and to be able to go deeper into the different aspects where there is a lack of information.**

***Response:*** *We thank the reviewer for bringing out the importance of this study.*

*************
